# Cannabis-Dependence Risk Relates to Synergism between Neuroticism and Proenkephalin SNPs Associated with Amygdala Gene Expression: Case-Control Study

**DOI:** 10.1371/journal.pone.0039243

**Published:** 2012-06-22

**Authors:** Didier Jutras-Aswad, Michelle M. Jacobs, Georgia Yiannoulos, Panos Roussos, Panos Bitsios, Yoko Nomura, Xun Liu, Yasmin L. Hurd

**Affiliations:** 1 Department of Psychiatry, Mount Sinai School of Medicine, New York, New York, United States of America; 2 Department of Pharmacology and Systems Therapeutics, Mount Sinai School of Medicine, New York, New York, United States of America; 3 Department of Psychiatry and Behavioral Sciences, Faculty of Medicine, University of Crete, Heraklion, Crete, Greece; 4 Department of Psychology, Queens College, Queens, New York, United States of America; 5 Institute of Psychology, Chinese Academy of Sciences, Beijing, People’s Republic of China; 6 Department of Neuroscience, Mount Sinai School of Medicine, New York, New York, United States of America; 7 James J Peters VA Medical Center, New York, New York, United States of America; The Scripps Research Institute, United States of America

## Abstract

**Background:**

Many young people experiment with cannabis, yet only a subgroup progress to dependence suggesting individual differences that could relate to factors such as genetics and behavioral traits. Dopamine receptor D2 (*DRD2*) and proenkephalin (*PENK*) genes have been implicated in animal studies with cannabis exposure. Whether polymorphisms of these genes are associated with cannabis dependence and related behavioral traits is unknown.

**Methodology/Principal Findings:**

Healthy young adults (18–27 years) with cannabis dependence and without a dependence diagnosis were studied (N = 50/group) in relation to *a priori-*determined single nucleotide polymorphisms (SNPs) of the *DRD2* and *PENK* genes. Negative affect, Impulsive Risk Taking and Neuroticism-Anxiety temperamental traits, positive and negative reward-learning performance and stop-signal reaction times were examined. The findings replicated the known association between the *rs6277 DRD2* SNP and decisions associated with negative reinforcement outcomes. Moreover, *PENK* variants (*rs2576573* and *rs2609997*) significantly related to Neuroticism and cannabis dependence. Cigarette smoking is common in cannabis users, but it was not associated to *PENK* SNPs as also validated in another cohort (N = 247 smokers, N = 312 non-smokers). Neuroticism mediated (15.3%–19.5%) the genetic risk to cannabis dependence and interacted with risk SNPs, resulting in a 9-fold increase risk for cannabis dependence. Molecular characterization of the postmortem human brain in a different population revealed an association between *PENK* SNPs and *PENK mRNA* expression in the central amygdala nucleus emphasizing the functional relevance of the SNPs in a brain region strongly linked to negative affect.

**Conclusions/Significance:**

Overall, the findings suggest an important role for Neuroticism as an endophenotype linking *PENK* polymorphisms to cannabis-dependence vulnerability synergistically amplifying the apparent genetic risk.

## Introduction

Marijuana (*Cannabis sativa*) is the illicit drug most commonly used in most Western societies [1]–[3]; it was consumed by at least 11.5% of individuals 12 years or older in the United States in 2010, even more so among teenagers (14%) and young adults (30%) [3]. Despite its common use, only a subset of teens and young adults using cannabis (25.4% and 19.0%, respectively) progress to abuse or dependence [4]. Such individuals become dependent on cannabis at a young age though cannabis typically has a delayed progression to dependence as compared to other drugs of abuse [5]. This difference in individual vulnerability has added to the heated debate as to whether cannabis is benign and should be legalized or a harmful drug whose status as an illegal substance should be maintained. Irrespective of the debate, cannabis-dependent individuals greatly outnumber those reporting dependence on other more addictive substances [3] and cannabis dependence carries a heavy burden, as it is associated with detrimental consequences on health [6]. Important research efforts have focused on cannabis use in association with specific disorders, such as psychosis, and among clinical populations with substantial comorbid psychopathology; but the larger cannabis-dependent population and the potential relevance of genetics and behavioral traits, such as reward sensitivity and neuroticism-anxiety, have been understudied.

Similar to other addictions, cannabis dependence is a complex disorder; thus, genetic interactions with factors such as behavioral traits and environmental conditions could contribute to addiction vulnerability. A growing number of family, twin and adoption studies have shown that cannabis-use disorder is influenced by heritability (30–80%) [7]–[15]. However, specific genetic determinants and behavioral factors remain unknown. In this study, we sought to examine factors relevant to cannabis-dependence risk by evaluating genetic polymorphisms of neural systems implicated in the actions of cannabis and to behavioral traits associated with addiction vulnerability.

Converging evidence obtained from animal and human brain studies has shown that early cannabis exposure selectively alters dopamine receptor D2 (*DRD2*) and proenkephalin (*PENK*) expression in the mesocorticolimbic system, disturbances that persist into adulthood and modulate drug-seeking behavior later in life [16], [17]. The sensitivity of striatal *PENK* and *DRD2* expression to delta-tetrahydrocannabinol (THC) raises the question as to whether genetic polymorphisms of these genes could in turn be associated with cannabis dependence. Moreover, the fact that these genes in the striatum are specifically colocalized in striatopallidal neurons, a critical component of the neuronal circuitry underlying inhibitory control, may have important implications to addiction vulnerability. While it is unknown whether genetic disturbances of *DRD2* or *PENK* could contribute to cannabis dependence, behavioral studies have demonstrated that SNPs in *DRD2* predict specific behavioral traits pertaining to reward sensitivity, inhibitory control and affect [18]–[20], endophenotypes known to be involved in addiction vulnerability. As such, we focused *a priori* on whether individual genetic differences of the *DRD2* and *PENK* genes associate with cannabis dependence and explored the possible mediation or moderation of intermediate endophenotypes in the genetic associations. Given the current lack of information as to the functional relevance of the *PENK* SNPs, we also assessed potential genotype relationships to *mRNA* expression levels in the postmortem human brain from another population sample.

## Methods

### Participants

Two hundred and eleven individuals were screened to enroll 100 participants meeting all eligibility criteria. The participants were between the ages of 18–27 and consisted of 50 controls and 50 subjects with a lifetime cannabis-dependence diagnosis (74.0% with current and 26.0% with past cannabis dependence). Eligible subjects were healthy males and females with no history of major psychiatric or medical disorders and no current or sustained past (more than one month) psychotropic medication. The recruitment of otherwise normal, non-treatment-seeking subjects aimed to have a study sample representative of the general cannabis population, as opposed to other cannabis-abuse populations with psychopathology and neuropsychiatric comorbidities. Cannabis-dependent subjects and controls had similar racial breakdowns and only differed in the percentage of Hispanic participants and daily cigarette use **(** **Table 1** **)**. Among controls, 66.0% reported cannabis use at least once.

### Description of Procedures Undertaken

Subjects were evaluated to obtain information regarding current health and sociodemographic characteristics. Exclusion of subjects with major psychiatric disorder was based on the Mini International Neuropsychiatric Interview [21]. Substance-abuse and -dependence diagnoses were determined using the Structured Clinical Interview for DSM-IV [22]. Exclusion criteria included major neurological/medical illness or taking systemic medication; history of head injury with loss of consciousness, neurological or cardiovascular disease, traumatic brain injury or any other condition that is likely to affect brain function; pregnancy; diagnosis or history of bipolar I or II disorder, ADHD, psychosis, pervasive developmental disorder, major affective disorder, motor tics or Tourette’s Syndrome, or seizure disorders; current or past use of psychotropic medication.

The cognitive probabilistic learning task involves a training phase in which participants learn to discriminate the feedback probabilities (positive feedback: green check mark; negative feedback: red cross) associated with different pairs of symbolic stimuli [18]. Participants learn from trial-and-error that symbol A was being rewarded with positive feedback in 80% of the trials, whereas symbol B was being rewarded in 20%. The distribution of positive-feedback probabilities for other pairs range from 70% versus 30% for Pair C–D, and 60% versus 40% for Pair E–F. Feedback is given for each trial. At the end of the training phase (128 trials), participants should learn that symbol A is the most favorable choice and the one most likely to get positive feedback, whereas symbol B is the least favorable one and should be avoided. During the testing phase, symbols A and B are paired with the other four symbols to create 8 new pairs for a total of 96 trials, and no feedback is given during the test. The results were tallied according to the proportions of trials in which symbol A was chosen over other symbols or symbol B was avoided when paired with other symbols. Performance on ‘’Choose A’’ pairs was used as a measure of positive-reinforcement learning, whereas performance on ‘’Avoid B’’ pairs reflected negative-reinforcement learning.

A version of the “stop-signal” Go/NoGo task was then administered to measure response inhibition. Subjects were asked to discriminate between letters “X” and “O” as quickly and accurately as possible. One third of the time they were told to withhold their responses for the “NoGo” trial when they heard a “stop” signal tone (a clear sound) presented at a variable delay after the letter was displayed. A staircase variation of the stop-signal-delay was used to keep the commission error (CE) rate at ∼50% for the “stop” trials, in line with previous studies [23], [24]. Stop-signal reaction time (SSRT), the mean length between the stop signal tone and the mean reaction time, measured the capacity of response inhibition.

Both tasks were administered approximately 2 hours after the subject’s arrival at the study site. Subjects were instructed to abstain from using alcohol or drugs (except nicotine) on the day of the testing session, which was not conducted if the participant showed any evidence of acute intoxication.

Positive and negative affective states were assessed with the (Positive and Negative Affect Schedule) PANAS [25] and personality traits were studied with the (Zuckerman-Kuhlman Personality Questionnaire) ZKPQ [26]. To confirm self-reported drug-use information, urine samples were screened for cannabis, along with other illicit drugs; all subjects also underwent a breathalyzer. None of the subjects screened positive for alcohol or any other drug than cannabis. 86.0% of the cannabis dependent individuals were positive for cannabis.

**Table 1:** Sociodemographic characteristics of the participants.

Characteristics	TotalN = 100 % (N)	Cannabis dependent subjectsN = 50 % (N)	ControlsN = 50 % (N)
Sex			
Male	67.0 (67)	74.0 (37)	60.0 (30)
Female	33.0 (33)	26.0 (13)	40.0 (20)
Age, mean (SD)	22.65 (2.78)	22.54 (2.57)	22.76 (3.00)
Marital status			
Married	9.0 (9)	10.0 (5)	8.0 (4)
Single or widowed	91 (91)	90.0 (45)	92.0 (46)
Ethnic background			
Hispanic or Latino*	41.0 (41)	52.0 (26)	30.0 (15)
African American	30.0 (30)	26.0 (13)	34.0 (17)
Caucasian	25.0 (25)	22.0 (11)	30.0 (15)
Asian	3.0 (3)	0.0 (0)	6.0 (3)
Education			
High school degree, GED, or higher	93.0 (93)	90.0 (45)	96.0 (48)
Less than high school degree or GED	7.0 (7)	10.0 (5)	4.0 (2)
Working status			
Working or studying	73.0 (73)	66.0 (33)	80.0 (40)
Unemployed	27.0 (27)	34.0 (17)	20.0 (10)
Lifetime cannabis use (yes)	83.0 (83)	100.0 (50)	66.0 (33)
Age at initiation of cannabis use(mean +/− SD)	16.01 (2.47)	15.66 (2.41)	16.55 (2.50)
Daily nicotine use***	29.0 (29)	54.0 (27)	4.0 (2)

Asterisk indicates significant difference between cannabis dependent and controls subjects. SD  =  Standard deviation.

* p<0.05;

*** p<0.001.

Genotype for cannabis dependence and control subjects was determined using an ABI 7900HT available at the Mount Sinai Quantitative PCR Shared Resource Facility. *DRD2* SNPs: *TaqIA* (rs1800497) is located ∼10 kb downstream of the *DRD2* and affects striatal *DRD2* receptor density [27], *rs6277* and *rs1076560* are associated with *DRD2* expression and cognitive/attentional performance [28]. *PENK* SNPs: *rs2576573, rs260999*7 and *rs6474063* were chosen based on pairwise linkage disequilibrium (LD) relationships (r^2^) of an r^2^ threshold of 0.8 and on haplotype data (www.hapmap.org) showing a minimum allele frequency of 0.10 in the population. The call rate was 100% and all genotypes examined conformed to Hardy-Weinberg equilibrium.

Fresh-frozen striatal and amygdala specimens were obtained from normal adult Caucasian subjects without head trauma from our brain bank collection [29] (collected at the Department of Forensic Medicine at Semmelweis University, Hungary). The specimens were collected under the guidelines approved by the local Human Ethical Committee within 24 hrs after death. The demographic characteristics of total subjects (N = 16) were: 36.8±3.0 years old; 13 males/3 females, 6.72±0.06 brain pH; postmortem interval 20.9±1.7 hrs; cause of death being cardiac failure (N = 12), viral infection (N = 2), pulmonary embolus (N = 1), electric shock (N = 1). Striatal samples were only available for 14 of the specimens.

*In situ* hybridization was performed (on 20 µm-thick cryosections as previously described to measure *PENK* mRNA expression levels) [29], [30]. The *PENK* riboprobe was an EcoRI/Pvu 792 bp fragment complementary to the full coding region of the *PENK* human gene [31]. Briefly, brain sections were hybridized with 20×10^3^ CPM/ µl [^35^S]-αUTP *PENK* riboprobe solution overnight at 55°C and following post-hybridization washes, exposed to Kodak Biomax MR film for 5 days. Optical density values measurements were taken over subregions of the striatum and the central amygdala and the values converted to DPM (disintegrations per minute)/mg by reference to co-exposed C^14^ standards (American Radiolabeled Chemicals, Inc., St. Louis, MO). DPM/mg values from duplicate slides were averaged.

### Ethics

All participants in this study gave written informed consent; Mount Sinai School of Medicine Institutional Review Board approved the study.

### Statistical Analysis

Independent t-tests and Chi-square tests were used to compare cannabis-dependent and control subjects according to sociodemographic characteristics. Association between genotype, behavioral traits and cannabis-use outcomes were analyzed by ANOVA and Pearson correlation, while group (based on cannabis-dependence diagnosis) and gene x group interaction effects were calculated using a general linear model. For each SNP, polymorphisms that were significantly associated with phenotype were evaluated for genotyping patterns of variants using the Fisher’s exact test. A reduction in unstandardized beta of >10% on cannabis dependence by the genetic factor after the potential mediator (e.g., Neuroticism) was included in the model, and was considered to be appreciably different and reported as a partial mediator. Additive interaction was evaluated based on the Rothman “index of synergism.” The presence/absence of an additive interaction was examined using an index, attributable proportion due to interaction (AP) and the 95% confidence interval (CI) estimated based on the Hosmer-Lemeshow CI estimation of interaction. AP exceeding zero indicates increased risk due to the two risk factors. Thus, the 95% CI for an AP that does not include a value of zero indicates statistical significance. To account for testing of multiple SNPs as predictors of cannabis dependence, we used the Bonferroni correction and, accordingly, all the tests used a 2-tailed α = 0.017 (0.05/3) significance level for each gene.

The mRNA expression (DPM/mg) data was normalized using natural logarithm. General linear stepwise regression analysis was used to evaluate genotype group differences with covariates: e.g., age, postmortem interval, brain pH, and sex. Statistical evaluations were assessed by using JMP 6.1 (SAS Institute, Cary, NC, USA) software.

## Results

**Figure 1:**
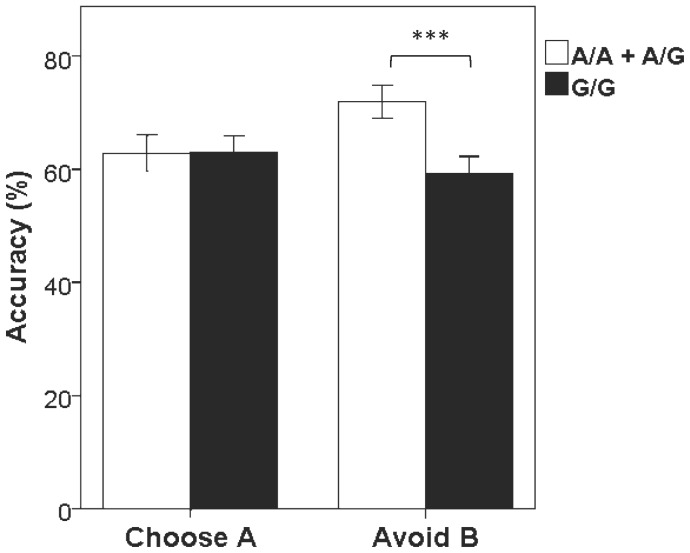
DRD2 SNP rs6277 in relation to the accuracy of choose A (positive reinforcement) and Avoid B (negative reinforcement) performance. ***, p<0.005 between the genotypes.

Previous behavioral studies have reported that DRD2 SNPs predict avoidance-based decisions in healthy subjects [18], [19], [32]. Results from the current population confirmed that negative reinforcement learning linked with the ability to avoid maladaptative choices was associated with the DRD2 rs6277 SNP (**Fig. 1**). There was an overall significant genotype difference, but there was no group or group x genotype interaction on Avoid B behavior. Subjects carrying the rs6277 A allele performed significantly better than G/G homozygous subjects on Avoid B pairs (p<0.005), but not for the positive Choose A performance. Other DRD2 and PENK SNPs examined did not influence reward learning performance in cannabis-dependent or control subjects, or in the whole sample. Inhibitory control as measured by SSRT was not modulated by any of the SNPs examined.

Evaluation of performance in the cognitive probabilistic learning tasks revealed that cannabis-dependent subjects and controls did not differ in their overall accuracy in the training phase (t = 0.15, p = 0.88) or during the testing session for reaction time (t = 0.075, p = 0.94). There was also no significant group difference for accuracy on either Choose A or Avoid B pairs (t = 0.066, p = 0.95, and t = 0.28, p = 0.78, respectively). On the stop-signal Go/NoGo task, the two groups also showed similar overall accuracy (t = 0.22, p = 0.83) and their SSRT did not differ significantly (t = 1.23, p = 0.22).

In evaluating genetic differences between cannabis and control subjects in regard to the DRD2 and PENK genes, it was observed that several DRD2 and PENK SNPs studied modulated cannabis-dependence outcomes (**Table 2**). A strong association was evident between the PENK SNP rs2576573 and cannabis-dependence diagnoses; 61.7% of cannabis-dependent subjects were A/G carriers, compared with 29.2% of controls (p<0.001). Similar findings were apparent for the other PENK SNP rs2609997 and the DRD2 SNP rs6277. Only the association between cannabis dependence and the two PENK SNPs remained significant after Bonferroni correction.

**Figure 2:**
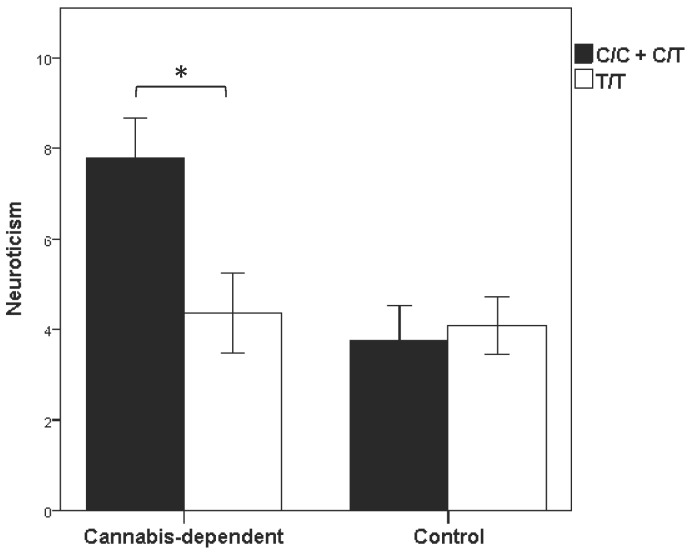
Neuroticism scores in relation to PENK rs2609997 SNP among controls, and in cannabis-dependent subjects. *, p<0.05 between the genotypes.

Subjects who were grouped based on a lifetime cannabis-dependence diagnosis (n = 50) or as controls (n = 50) differed in relation to affect and temperamental traits as reported on the PANAS and ZKPQ. Our study focused on Impulsive Risk Taking and Neuroticism-Anxiety subscales of the ZKPQ which we used as a temperamental proneness to poor inhibitory control as well as negative emotional traits (negative affect and anxiety), respectively. Cannabis-dependent individuals reported more negative affect on the PANAS (p = 0.013) and higher scores on the Neuroticism (p = 0.004), Impulsivity (p = 0.0002) and Aggression-Hostility (p = 0.0008) subscales of the ZKPQ, as well as a lower mean score on Activity-Energy (p = 0.049). Significant associations were detected between genetic polymorphisms and temperamental traits. Neuroticism was associated with rs6277 (p<0.05), rs2576573 (p<0.01) and rs2609997 (p<0.05), as shown in table 3. There was a significant cannabis-dependence group effect (F = 6.2, p = 0.02) and SNP x cannabis-dependence interaction (F = 4.7, p = 0.03) for the PENK SNP rs2609997, as this SNP was significantly associated with Neuroticism in cannabis-dependent subjects (p<0.05), but not in control subjects (p = 0.8; **Fig. 2**). None of the SNPs were associated with Impulsivity.

**Table 2:** Genotype distributions in cannabis dependent subjects and controls for the SNPs studied.

SNPs	Genotype	Cannabis dependentN (%)	ControlsN (%)
D2 rs6277*	A/A	2 (4.0)	10 (20.0)
	A/G	22 (44.0)	15 (30.0)
	G/G	26 (52.0)	25 (50.0)
D2 rs1076560	A/A	1 (2.0)	1 (2.0)
	A/C	13 (26.0)	14 (28.0)
	C/C	36 (72)	35 (70.0)
D2 rs1800497	A/A	5 (10.0)	3 (6.0)
	A/G	16 (32.0)	23 (46.0)
	G/G	29 (58.0)	24 (48.0)
PENK rs2609997**	C/C	6 (12.0)	4 (8.0)
	C/T	25 (50.0)	12 (24.0)
	T/T	19 (38.0)	34 (68.0)
PENK rs2576573***	A/A	6 (12.8)	4 (8.3)
	A/G	29 (61.7)	14 (29.2)
	G/G	12 (25.5)	30 (62.5)
PENK rs6474063	C/C	0 (0.0)	3 (6.0)
	C/T	15 (30.0)	13 (26.0)
	T/T	35 (70.0)	34 (68.0)

N = 100, except for PENK rs2576573 for which 5 subjects were excluded due to genotyping failure. Asterisks indicate a significant SNP x group (cannabis dependence diagnosis vs. control) effect.

* p<0.05,

** p<0.01,

*** p<0.001, uncorrected.

**Figure 3:**
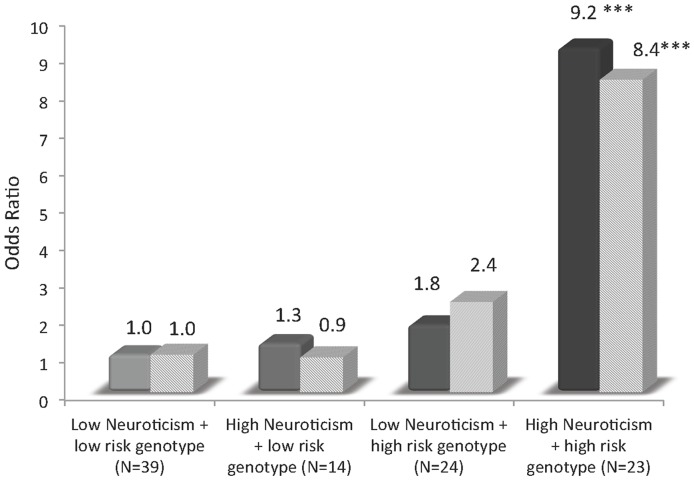
High risk genotype  = C/C + C/T for rs2609997 or A/A + A/G for rs2576573; low risk genotype  = T/T for rs2609997 or G/G for rs2576573. ***p<0.01; ****p<0.001.

Mediation models were subsequently examined to determine whether behavioral traits mediated the genetic association with cannabis outcomes. Neuroticism was a notable mediator of the association between rs6277, rs2576573, and rs2609997 and cannabis dependence; 15.3% to 19.5% association between the gene and cannabis dependence was explained by Neuroticism. None of the inhibitory control, reward learning or impulsive temperamental traits met criteria as mediators of the associations between any SNPs and cannabis dependence outcomes. We further examined the potential interaction (moderation) effect of having a genetic and temperamental risk focusing on Neuroticism, which was the most strongly associated with cannabis dependence. We categorized Neuroticism by median score into high and low Neuroticism groups and evaluated four groups: high genetic risk and high Neuroticism; high genetic risk and low Neuroticism, low genetic risk and high Neuroticism; neither risk (reference group). **Figure 3** shows a clear evidence for additive interaction with AP being 0.67 (95% CI, 0.62–0.72), indicating 67% increased risk is due to synergy. Relative to the reference group, those with high Neuroticism (OR = 1.3, p = .85) or an at-risk SNP (PENK rs2609997; OR = 1.8, p = .26) did not have notable increased risk, but those with high Neuroticism and the at-risk SNP had an over 9-fold increased clinical diagnosis of cannabis dependence (OR = 9.2, p = .0007). A similar additive interaction was detected for the PENK rs2576573 [AP = 0.72, (95% CI, 0.58–0.86)]. Relative to the reference group, those with both at-risk SNP (PENK rs2576773) and high Neuroticism had an over 8-fold increased clinical diagnosis of cannabis dependence (OR = 8.35, p = .001), compared to those with only Neuroticism (OR = 0.93, p = .99) or the at-risk SNP (OR = 2.41, p = .27). It was not possible to study the DRD2 rs6277 SNP due to the high overlap between high Neuroticism and subjects with the G allele.

**Table 3:** Neuroticism score in relation to SNPs studied.

SNPs	Genotype	Neuroticism (SD)
D2 rs6277*	A/A	3.50 (2.68)
	A/G	6.62 (4.78)
	G/G	4.63 (4.23)
D2 rs1076560	A/A	9.00 (8.49)
	A/C	5.44 (4.56)
	C/C	5.04 (4.27)
D2 rs1800497	A/A	5.75 (5.12)
	A/G	4.87 (4.40)
	G/G	5.42 (4.37)
PENK rs2609997*	C/C	4.50 (3.69)
	C/T	6.92 (5.01)
	T/T	4.19 (3.75)
PENK rs2576573**	A/A	4.50 (3.69)
	A/G	6.98 (4.86)
	G/G	3.67 (3.52)
PENK rs6474063	C/C	5.33 (4.73)
	C/T	6.21 (4.49)
	T/T	4.83 (4.37)

Given that most cannabis-dependent subjects smoked cigarettes and the documented differences between cigarette smokers and non-smokers in terms of Neuroticism [33], we evaluated whether nicotine use may be the factor driving behavioral specificity in cannabis-dependent subjects. In our sample, there were no differences as to temperamental traits, affect, reward learning, inhibitory control and SNPs between cannabis-dependent subjects who smoked cigarettes daily compared to those who did not. To further address this issue, we also examined *PENK* SNPs in another population (homogenous Caucasian Greek army conscripts; **Table 4**) in which personality traits had been obtained (N = 559, 247 smokers and 312 non-smokers; mean age = 23.4 years), as described previously [34]. Cigarette smoking without cannabis use was associated with Neuroticism (p = 0.02), but it was not significantly related to the *PENK* SNPs (*rs2609997, rs2576573*). Interestingly, although the cannabis-dependence diagnosis was not assessed in this population, an additive-interaction effect between Neuroticism and both *PENK* SNPs predicted the history of cannabis use (*rs2609997*, p = 0.01; *rs2576573*, p = 0.02).

**Table 4:** Sociodemographic characteristics and genotype distribution for the proenkephalin SNPs studied in the Caucasian Greek cohort.

	Smokers(N = 247)	Non-smokers(N = 312)	P value
Age, mean (SD)	23.72 (4.13)	23.08 (4.47)	0.084
Education, mean (SD)	15.62 (2.68)	15.23 (2.85)	0.097
Neuroticism score [Eysenck Personality Questionnaire] (SD)	10.83 (4.9)	9.78 (5.07)	0.005
**SNPs**	**Genotype**	**Smokers** **N (%)**	**Non-smokers** **N (%)**
rs2576573#	G/G	83 (33.6)	103 (33.0)
	G/A	117 (47.4)	137 (43.9)
	A/A	47 (19.0)	71 (22.8)
rs2609997##	A/A	76 (30.8)	93 (29.8)
	G/A	120 (48.6)	140 (44.9)
	G/G	50 (20.2)	76 (24.4)

All subjects were healthy control males from a highly homogenous Caucasian population of army conscripts. Minor allele frequency was 0.44 (rs2576573) and 0.46 (rs2609997). Hardy-Weinberg equilibrium for the full population: χ^2^ = 3.2 (p = 0.07) rs2576573 and 1.8 (p = 0.18) for rs2609997. SD: standard deviation.

# one non-smoker with missing genotype;

## one smoker and two non-smokers with missing genotype.

**Figure 4:**
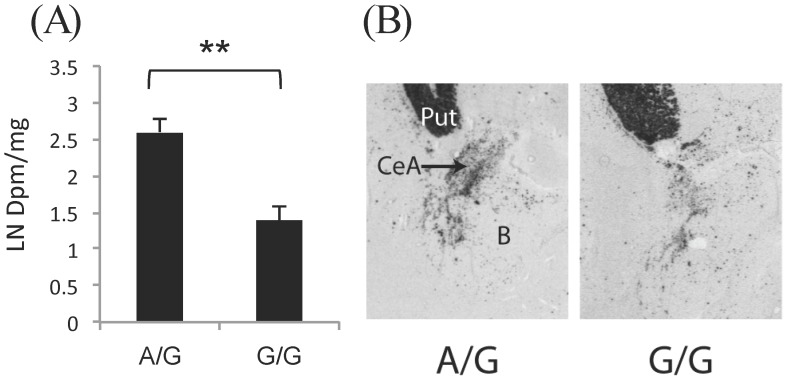
PENK mRNA distribution (A) and expression levels (B) in the amygdala of control subjects with the A/G and G/G genotype for the rs2576573 SNP. Expression levels (mean ± SEM) are denoted as natural log of the DPM/mg values. **p<0.01.

Very limited information is known about the neurobiological relevance of mutations of the PENK gene. Thus, given that the current PENK SNPs studied are within noncoding regions of the gene, we examined their potential relationship to PENK mRNA expression levels in the postmortem striatum and amygdala, regions highly implicated in emotional regulation in normal human subjects. PENK mRNA is expressed throughout the striatum and predominantly in the central nucleus of the amygdaloid complex (**Figure 4**). A strong significant association was detected between the PENK SNPs and mRNA expression in the central amygdala (rs2576573 F1,15 = 13.43, p = 0.003). Overall, control subjects with the A allele of the rs2576573 SNPs had higher PENK expression (**Figure 4**). In the striatum, PENK mRNA expression was significantly influenced by age and only a trend-genotype effect was detected in the nucleus accumbens core (F1,10 = 3.778, p = 0.084). A gene-dose effect was also evident for the rs2609997 SNP in the central amygdala (F2,15 = 5.577, p = 0.018) with C/C subjects having higher PENK mRNA expression, but there were few homozygous subjects, so this SNP could not be fully evaluated in the postmortem population.

## Discussion

Although cannabis does not have high abuse liability as other drugs, it is clear that some individuals are more susceptible to becoming dependent on cannabis even at a young age. Decreased negative reward/avoidance behavior conferred by *DRD2* individual genetic differences has been hypothesized to account for such vulnerability. While we replicated previous findings showing a role of *DRD2* in negative reward learning [18], [19], [32], cannabis-dependence vulnerability did not appear to be driven by this association. Instead, the results suggest for the first time to our knowledge that there are fundamental biological differences driven by genetic impairments in the opioid system in cannabis-dependent individuals compared to those who used the drug but did not become dependent. The study further revealed that Neuroticism, in contrast to other traits characteristic of cannabis-dependent subjects or compared to reward learning and inhibitory control performance, significantly mediates the association between functional SNPs of *PENK* (which we now know are associated with mRNA expression levels in regions relevant to negative emotional states) and cannabis dependence. The finding that cannabis dependence is significantly enhanced in a synergistic fashion in individuals with both high Neuroticism and risk genotypes emphasizes the potential important synergistic contribution of negative emotional traits and genetics to vulnerability.

Prevailing theories postulate that addiction vulnerability is linked to impairment of reward sensitivity and/or inhibitory control. Recently, genetic polymorphisms have been used to dissect potential striatal dopamine-receptor involvement in decision-making in relation to positive and negative reinforcement outcomes [18], [19], [32]. Our data supports an association of the *DRD2* SNP *rs6277* with negative reward performance, but not for other *DRD2* SNPs studied. Other investigations [32] have also indicated a differential effect of the *DRD2* SNPs on behavior that may relate to the role of these SNPs in modulating *DRD2* functions (i.e., *DRD2* binding, density). The ability to replicate previous studies of the *DRD2 rs6277* SNP with avoidance learning emphasizes the validity of our study population. Moreover, the overall lack of association between reinforcement learning and inhibitory control to cannabis dependence either directly or as mediators suggest that although these behavioral traits are important to various aspects of addiction, they do not appear to contribute to cannabis-dependence vulnerability to the same extent as negative emotional traits.

On the other hand, the central role of Neuroticism in the association between *PENK*/*DRD2* genes and cannabis-use outcomes in our sample has important implications. Both clinical reports and research data have suggested that coping with stress and negative mood states is a common motive for use among heavy abusers [35], which would be consistent with self-medicating subthreshold anxiety and negative affect induced by *PENK* dysfunction. Interestingly, previous studies that have addressed the self-medication hypothesis showed that cannabis is more likely to exacerbate mood symptoms than to alleviate them [36]. Cannabis exposure and negative affect may thus interact in a complex way within a vicious cycle where cannabis may be used to cope with subthreshold symptoms, but paradoxically further increase them in the long term. That our population consisted of non-depressed subjects underscores the fact that subthreshold symptoms that are not captured by DSM-IV may well have an impact on the emergence and course of a clinically significant disorder such as cannabis dependence.

Another important finding is the role of *PENK* SNPs in predicting cannabis dependence. The vulnerability conferred by *PENK* (and to a lesser extent *DRD2*) SNPs may reflect disturbances of specific neurobiological systems common to these genes. Both *DRD2* and *PENK* genes are strongly expressed in the striatum and amygdala, brain regions highly relevant to addiction disorders. A vast literature has emphasized the role of *DRD2* in addiction [37]; but significant evidence also suggests *PENK* involvement in mood/reward regulation and anxiety that are often correlated with alterations of the mesocorticolimbic system [38]–[40] and the striatopallidal circuitry in aversive behavior [41]. The amygdala plays a particularly prominent role in negative mood states and enkephalinergic neurons in the central amygdala are known to be critically involved in anxiety and stress responsivity [38], [40]. Interestingly, the association detected in the current study between *PENK* SNPs and *mRNA* expression was most pronounced in the central amygdala. The *rs2576573* A allele, observed to be more frequent in cannabis subjects, was associated with higher *PENK mRNA* expression in the central amygdala of the postmortem population in control subjects. This would seem contrary to predictions based on animal studies since upregulation of amygdala *PENK mRNA* expression predicts heightened anxiolytic responses and cannabis subjects exhibited more Neuroticism/anxiety. However, it is important to note that it was not possible to study *mRNA* expression in the brains of cannabis users, so whether the same relationship exists between the A *rs2576573* allele and mRNA expression in cannabis-dependent subjects needs to be investigated. Nevertheless, the current observation does document for the first time that *PENK mRNA* expression, particularly in the amygdala, directly associates with these polymorphic noncoding *PENK* SNPs. The fact though that the entire *PENK* gene is in strong LD suggests that the causative mutation still remains to be identified.

There are a number of limitations that should be considered when evaluating our findings. The lack of association observed regarding some SNPs, inhibitory control and negative-reward sensitivity could be related to the study’s design. The small sample size may have limited our ability to detect genetic effects in subgroups based on dependence diagnoses. For example, few cannabis-dependent individuals carried the *A* allele of the *DRD2 rs6277*, so it was not possible to explore behavioral traits in relation to this SNP. Another factor to consider is the cross-sectional approach of this study. The acute neurocognitive effects of cannabis might have affected task performance. While subjects had at least 2 hours of observed abstinence before performing the tasks, we cannot exclude the contribution of such confounding factors and our results should be replicated in settings where chronology of recent use may be better monitored. The fact that our sample was drawn from a population without significant psychiatric comorbidity also opens up the question as to whether these results may be generalizable to other comorbid subgroups. Replication of our results in a larger cohort will also be important to address potential stratification effects. Although not specific to cannabis dependence, results from the Greek Caucasian population suggest that the association between the *PENK* SNPs and Neuroticism could be replicated and generalized to other populations. An additional issue to address in interpreting the data is that a large percentage of the cannabis group also smoked cigarettes; thus, nicotine withdrawal could be a potential confound to the Neuroticism trait ascribed to cannabis-dependent subjects. However, study participants did not abstain from nicotine on the day of testing and behavioral traits were determined within an hour of the subject’s arrival to reduce potential withdrawal complications. Moreover, cigarette smoking was not associated with *PENK* SNPs in the large Caucasian population; cigarette smoking is thus unlikely to explain the genetic findings in our subgroup of cannabis-dependent subjects who also smoked cigarettes.

Overall, our results further support the role of *DRD2* in negative reward learning, and suggest a central role for Neuroticism as an endophenotype linking *PENK* polymorphism to cannabis-dependence vulnerability synergistically amplifying the apparent genetic risk. Future studies are needed to correlate neurobiological outcomes with behaviors in animal models and other human populations. Nonetheless, this study suggests that subthreshold mood and anxiety symptoms that do not meet criteria for a DSM-IV disorder may have serious clinical implications. Prevention and early intervention approaches that focus on coping strategies among young individuals genetically prone to Neuroticism may prove to be helpful, and should be specifically examined in such populations.
